# Use of HPLC-MS to Determine the Loss of Metabolites in Apple Juices under Different Storage Conditions

**DOI:** 10.3390/foods12152822

**Published:** 2023-07-25

**Authors:** Jan Juhart, Aljaz Medic, Jerneja Jakopic, Robert Veberic, Metka Hudina, Franci Stampar

**Affiliations:** Department of Agronomy, Biotechnical Faculty, University of Ljubljana, 1000 Ljubljana, Slovenia; juhartj@gmail.com (J.J.); jerneja.jakopic@bf.uni-lj.si (J.J.); robert.veberic@bf.uni-lj.si (R.V.); metka.hudina@bf.uni-lj.si (M.H.); franci.stampar@bf.uni-lj.si (F.S.)

**Keywords:** *Malus domestica* Borkh., organic acids, sugars, phenolic compounds, pasteurized apple juice, storage, color, processing, HPLC-MS identification

## Abstract

The focus of this experiment was to compare the color and metabolic profile of apple juice from the red-fleshed cultivar ‘Baya Marisa’ with the white-fleshed cultivar ‘Golden Delicious’. The changes in the phenolic compounds, organic acids, and sugar content during high-temperature short-time pasteurization and after one year of storage under different storage conditions were analyzed. A total of 26 individual phenolic compounds were identified and quantified. The total analyzed phenolics content (TAPC) decreased after pasteurization of the juices of both cultivars. The TAPC of fresh ‘Baya Marisa’ juice after pasteurization increased or remained the same compared to one-year stored ‘Baya Marisa’ juice, depending on the storage method. The sucrose content of the apple juice of both cultivars remained the same after pasteurization; interestingly, it decreased significantly after one year of storage, while the fructose and glucose content remained the same after pasteurization and increased significantly after one year of storage for both cultivars.

## 1. Introduction

The phytochemical composition of apples, which includes the content of organic acids, sugars, and phenolic compounds, is known to depend on the growing season, cultivars, climatic conditions, and storage methods [1]. Apple fruits (*Malus domestica* Borkh.) are renowned for their abundant phenolic compound content, which has been associated with various health benefits for humans [2]. Six main groups of phenolic compounds are found in fresh apple fruit: hydroxycinnamic acids, dihydrochalcones, flavanols, flavonols, anthocyanins [3], and hydroxybenzoic acids [4].

Apples are highly popular among consumers because of their flavor and wide availability throughout the year, whether consumed fresh or in processed forms. A large share of apples is consumed fresh. However, apple fruits are also processed into products such as apple juice (cloudy and clear), dehydrated apple slices, apple chips—dry apple slices, vinegar, and fermented cider [4]. The content of the phenolic compounds during processing is determined using various processing techniques [4]. Fruit is processed into juices in several steps: pressing, filtering, and heating during pasteurization to minimize microbial spoilage and increase the shelf life of fruit juices [5,6].

Usually, the phenolic composition of fruits is influenced by environmental conditions and genetic factors, while storage and processing can modify the phenolic composition due to oxidative reactions [7].

The bioavailability of the phenolic compounds in apples, which are subjected to different processing techniques, may have a positive (release) or negative (degradation) effect on the bioactive compounds in apples [8].

The color of beverages is a crucial attribute because it is the first factor that consumers perceive on store shelves, and it affects the attractiveness of products [9]. The demand for red beverages is increasing because they are associated with healthy properties due to pigments such as anthocyanins. However, anthocyanins are an unstable group of phenolic compounds that are degraded during processing and storage, resulting in a brownish color [9].

The activity of two groups of enzymes, polyphenol oxidase (PPO) and peroxidase (PDO), is important for the color of fruit juices [10]. Cloudy apple juices may experience enzymatic browning, which is a problem because it affects the nutritional and sensory properties of fruit juices [10]. Browning results from the degradation of phenolic compounds to colored quinones (substrate for further reactions leading to pigment formation) as a result of oxidation-reduction enzymes [10].

Consumer interest in foods with preserved functional and nutritional properties is increasing [11]. However, to preserve apple juice and extend its shelf life, apples must undergo thermal (pasteurization or sterilization) or non-thermal (high pressure processes) treatments. Thermal treatments are the most widely used technique for preserving juices. The temperatures of thermal treatments are generally 70–90 °C for pasteurization, 110–120 °C for sterilization, and 140–160 °C for ultrahigh-temperature treatment [12]. Pasteurization is a highly effective technique for prolonging the lifespan of fruit juices, as it mitigates the detrimental impact of enzymes on the juice throughout the storage process. Processing and storage affect the brightness of cloudy apple juices, as fruit juices have been reported to darken during storage, possibly due to browning caused by the enzymes from two groups, polyphenol oxidase and peroxidase [10].

The objective of this research was to conduct a comprehensive comparison of the metabolite composition (including the phenolic compounds, sugars, and organic acids) between the red-fleshed apple cultivar ‘Baya Marisa’ and the traditional white-fleshed apple cultivar ‘Golden Delicious’ before and after pasteurization. In addition, the metabolites were also measured after one year of storage of the pasteurized apple juice in a refrigerator, with samples that were either unexposed to light or exposed to light. This research focuses on the analysis of the phenolic compounds, sugars, organic acids, and color in both fresh unpasteurized and fresh pasteurized apple juice. Additionally, it examines the characteristics of pasteurized apple juice stored for one year from ‘Baya Marisa’ and ‘Golden Delicious’ apple cultivars. These findings provide valuable insight for future studies exploring the metabolic profile of processed apples and the impact of thermal treatment on apple juice. The results are also important for consumers, as they provide information on which storage method is best in terms of metabolite and color loss and how different storage methods affect apple juice quality (metabolites, color).

## 2. Materials and Methods

### 2.1. Plant Material and Sample Preperation

The intensive orchard where both apple cultivars, ‘Baya Marisa’ and ‘Golden Delicious’, were harvested is located in the southeastern area of Slovenia, in Zdole (45°98′02″ N; 15°52′07″ E; 307 m.a.s.l.). In the orchard, both cultivars of apple trees were grafted onto M9 rootstocks and spaced at intervals of 3.2 m × 0.9 m and were in their fifth growing season. The rows in the orchard were positioned in a north–south direction. The apple trees were subjected to standard integrated pest management practices. The harvesting of ‘Golden Delicious’ took place on 8 September 2022, followed by the harvest of ‘Baya Marisa’ on 16 September 2022. Subsequently, the apples were transported to a laboratory for analysis and subsequent processing.

**Figure 1:**
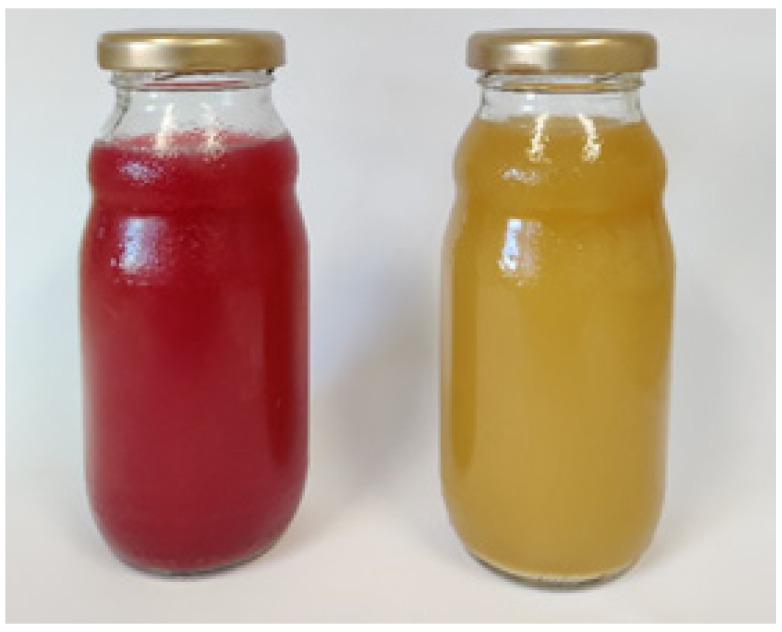
Apple juice in 100 mL glass bottles of cultivars ‘Baya Marisa’ (**left**) and ‘Golden Delicious’ (**right**).

The analyses were performed in the laboratory of the Department of Agronomy, Biotechnical Faculty, University of Ljubljana (Slovenia), where 30 apples, 15 per cultivar, were randomly selected as replicates and further processed (juice color, organic acids, sugars, and phenolic compounds of unpasteurized and pasteurized apple juice were the subjects of this study). The cores of the apples were removed, and the apples were then pressed in a VitaExtract Slow Juicer (Bosch, Gerlingen, Germany) and filtered through MN615 Ø 150 mm filter paper (Macherey-Nagel). The yield of each apple cultivar was about 60%, 1.8 L per cultivar. There were three replicates per treatment, with each bottle representing a biological replicate (Figure 1). Three bottles of 100 mL juice of both apple cultivars were analyzed immediately after pressing. The remaining bottles were pasteurized. Three bottles per cultivar were analyzed immediately after pasteurization, while the rest were stored under standard household conditions (in a refrigerator, at room temperature unexposed to light, and at room temperature exposed to light). Pasteurization was performed by the widely known and used conventional VHTST (very high-temperature short-time) method, 85 °C for 30 s [13]. The apple juice of both cultivars was cloudy because it was not treated with the enzyme pectinase, which causes depectinization, the main substance responsible for apple juice cloudiness [14]. The unpasteurized juice was not stored and measured because it would be spoiled by microbial activity after one year of storage.

### 2.2. Apple Juice Color Measurements

The color of the apple juice (chromatic parameters) was measured by spectrophotometric determination (Perkin-Elmer, UV-vis Lambda Bio 20 spectrophotometer, Waltham, MA, USA) for the immediately pressed unpasteurized, pasteurized apple juice, and stored pasteurized apple juice of both cultivars (refrigerated, unexposed to light, and exposed to light). There were three replicates per treatment, with 100 mL representing one biological replicate. The tonality (To) and color intensity (CI) were measured according to a previous method [15]. The color intensity was calculated using the sum of the absorbance values at 420, 520, and 620 nm in a quartz cuvette with a 10 mm optical path. The tonality indicates the ratio between yellow–orange (420 nm) and red (520 nm) pigments. The CI, To, % yellow, % red, and % blue were calculated using the following formulas:

CI = A420 + A520 + A620(1)

TO = A420/A520(2)

% yellow = (A420/CI) × 100(3)

% red = (A520/CI) × 100(4)

% blue = (A620/CI) × 100(5)

### 2.3. Analysis of Sugars, Organic Acids, and Phenolic Compounds

For the analysis of the sugars, organic acids, and phenolic compounds, the method described in previous protocols was used [16,17,18]. All the treatments, i.e., the various juices, were filtered using 0.2 µm cellulose filters (Chromafil Xtra MV-20/25; Macherey-Nagel, Düren, Germany) into vials for the subsequent analysis of the sugars and organic acids. For the analysis of the phenolic compounds, 0.2 µm polyamide filters (Chromafil AO-20/25; Macherey-Nagel, Düren, Germany) were used for filtration. The vials containing the filtered samples were stored at −20 °C until further analysis of the organic acids, sugars, and phenolic compounds. The ascorbic acid was extracted in a 1:1 ratio, whereby 1 mL of apple juice was mixed with 1 mL of 2% metaphosphoric acid.

### 2.4. HPLC Analysis of Organic Acids and Sugars

Commercial standards and Vanquish, a UHPLC system from Thermo Scientific (San Jose, CA, USA), were used to identify the individual organic acids and sugars. To separate the individual sugars, a Rezex RCM monosaccharide column from Phenomenex (Torrence, CA, USA) was used and operated at a temperature of 85 °C and a flow rate of 0.6 mL/min. The eluted carbohydrates were measured using a refractive index detector (Refractomax 520, Idex health and science KK 5-8-6, Nishiaoki Kawaguchi, Japan) in accordance with the methodology described by Medic et al. [19]. For the identification of the organic acids, a Rezex ROA column from Phenomenex operated at 65 °C and a UV detector set at 210 nm were used, following the same UHPLC system and methodology previously described by Medic et al. [20]. The ascorbic acid analysis was conducted at 20 °C with a UV detector at 210 nm using the same UHPLC system, based on the approach outlined by Bizjak et al. [21]. The mobile phase for the sugar analysis consisted of double-distilled water, while the mobile phase for the organic acids analysis comprised 4 mM of sulfuric acid in double-distilled water. The content of the organic acids and sugars was expressed in milligrams per liter (mg/L) of juice.

### 2.5. HPLC-MS Analysis of Phenolic Compounds

The analysis of the phenolic compounds was conducted using a UHPLC system (Thermo Scientific; San Jose, CA, USA). The diode array detector was configured at specific wavelengths: 350 nm for flavonols, 530 nm for anthocyanins, and 280 nm for other phenolic compounds. The experimental conditions adhered to the established protocols [18], and a 20 µL injection volume was utilized. The spectra were recorded within the range of 200 to 600 nm. A C18 column (Gemini; 150 × 4.60 mm, 3 µm; Phenomenex, Torrance, CA, USA) was employed for compound separation, operating at a temperature of 25 °C. The phenolic compounds were identified through tandem mass spectrometry (MS/MS; LCQ Deca XP MAX; Thermo Scientific, Waltham, MA, USA), utilizing heated electrospray ionization in positive ion mode for detecting the anthocyanins and negative ion mode for detecting the hydroxycinnamic acids, dihydrochalcones, flavanols, and flavonols. For analysis, MS scans ranging from 50 to 2000 *m*/*z* were acquired. The data acquisition was facilitated by Xcalibur 2.2 software (Thermo Fischer Scientific Institute, Waltham, MA, USA). Whenever available, external standards were employed for both identification and quantification. In cases where standards were not available, literature data and fragmentation patterns were utilized to identify the unknown compounds.

The total analyzed phenolic content (TAPC) represents the cumulative amount of all the identified phenolic compounds. Both the TAPC and individual phenolic compounds are expressed in milligrams per liter (mg/L) of juice.

### 2.6. Chemicals

The Milli-Q water purification system (Millipore, Bedford, MA, USA) was used to purify bi-distilled water for sample preparation and analysis. Metaphosphoric acid was purchased from Sigma-Aldrich (Steinheim, Germany).

The standards employed for the analysis included the following compounds: caffeic acid, citric acid, cyanidin-3-*O*-galactoside, (-) epicatechin, ferulic acid, fructose, fumaric acid, glucose, malic acid, sorbitol, sucrose, phloridzin, *p*-coumaric acid (obtained from Fluka Chemie GmbH, Buchs, Switzerland), chlorogenic acid, cryptochlorogenic acid, protocatechuic acid (purchased from Sigma-Aldrich Chemie GmbH, Steinheim, Germany); cyanidin-3-*O*-arabinoside and peonidin-3-galactoside, malvidin-3-glucoside, and malvidin-3-pentoside (sourced from Apin Chemicals, Abingdon, UK).

### 2.7. Statistical Analysis

The data collection was conducted using Microsoft Excel 2016. The statistical analysis was carried out using R commander. Significant differences among the data were determined through one-way analysis of variance (ANOVA) with Tukey tests. To establish significance (*p* < 0.05), the statistical means were calculated with a 95% confidence level. All the data are presented as means ± standard errors (SE).

## 3. Results and Discussion

### 3.1. Juice Color

One of the most important visual characteristics of apple juice is the appealing color, which is represented by the composition of the percentage of red, yellow, and blue that contribute to the overall color appearance, while the total amount of color is represented by the color intensity (Table 1).

The color intensity of the red-fleshed ‘Baya Marisa’ decreased after pasteurization, while the color intensity of the white-fleshed ‘Golden Delicious’ did not change after pasteurization. After one year of storage in a refrigerator, unexposed to light, and exposed to light, the color intensity of both apple juices decreased. ‘Baya Marisa’, which was stored under exposure to light, showed a slightly decreased color intensity compared to the pasteurized and unpasteurized apple juice of the same cultivar, but it was significantly higher than the color intensity of the apple juice stored in the refrigerator and unexposed to light. The highest color intensity of the stored samples of ‘Golden Delicious’ juice was in the refrigerator, and the lowest was in the samples unexposed to light. The percentage of yellow color in ‘Golden Delicious’ decreased after pasteurization and after one year of storage in the refrigerated samples, the samples unexposed to light, and samples that were exposed to light. It should be noted that the percentage of yellow of the stored apple juice of the ‘Golden Delicious’ cultivar was highest in the unexposed to light samples, but still slightly lower than the fresh pasteurized and unpasteurized apple juice of the same cultivar. The percentage of yellow in the red-fleshed ‘Baya Marisa’ apple juice increased after one year of storage, with the percentage of the samples unexposed to light being the highest. The percentage of red color increased slightly after pasteurization but decreased after one year of storage, with the lowest percentage of red occurring in the apple juice of ‘Baya Marisa’ in closed storage. The percentage of red color increased in the ‘Golden Delicious’ apple juice after pasteurization and continued to increase after one year of storage, with the highest percentage occurring in ‘Golden Delicious’ apple juice stored in a refrigerator. The percentage of blue color of the apple juice of ‘Baya Marisa’ increased slightly after pasteurization and was the highest in apple juice exposed to light, while it was lowest in apple juice of the same cultivar unexposed to light. In ‘Golden Delicious’ apple juice, the blue content did not change after pasteurization, but increased after one year of storage, with the highest blue content in the apple juice of ‘Golden Delicious’ stored in a cool place. The tonality was highest in ‘Baya Marisa’ apple juice stored in the refrigerator, while in ‘Golden Delicious’, the unpasteurized and freshly pasteurized apple juice had the highest tonality, i.e., the highest proportion of yellow color and the lowest proportion of red color.

Previous studies have reported comparable findings, as well as consistent results from Petrovic et al. [22], where an increase in the percentage of yellow color and a loss of red color were observed in apple liqueur after the evaluated storage time. The decrease of red color in the red-fleshed ‘Baya Marisa’ could be due to the precipitation of the anthocyanin pigments or the degradation of anthocyanins by oxidation [23]. On the other hand, the observed decline in the red color of both apple juices following storage may be attributed to a potential process involving the oxidation of chlorogenic acid in the presence of oxygen, leading to the formation of the corresponding quinone. This quinone then interacts with anthocyanins, resulting in the production of brown condensation products [24]. In our study, the HTST technique was used for pasteurization; however, some studies report that the loss of red color (degradation of anthocyanins) can be prevented by lower temperatures combined with a short processing time, since anthocyanins are more stable at lower temperatures [24]. Since the red color in the apple juice was more intense in the pasteurized apple juices from ‘Baya Marisa’ stored at lower temperatures (in a refrigerator), this suggests a more rapid degradation of anthocyanins at higher temperatures [24].

### 3.2. Content of Sugars and Organic Acids

The sugar and organic acid composition, as well as their ratio, play a crucial role in determining the quality and taste of fruit juices [25]. In the apple juice of these two cultivars, four different sugars and three different organic acids were identified. Table 2 presents the content of the sugars and organic acids in freshly pressed unpasteurized apple juice, freshly pasteurized apple juice, and pasteurized juice stored for one year in the refrigerator, both unexposed to light and exposed to light.

The sucrose content of the fresh and pasteurized juice of ‘Baya Marisa’ was similar, while the sucrose content decreased significantly after one year of storage in the refrigerator, unexposed to light, and exposed to light, with the content being the same among the treatments. The sucrose content of the fresh pasteurized and unpasteurized apple juice of ‘Golden Delicious’ was the same. After one year of storage, the sucrose content continued to decrease, with the highest content occurring in apple juice stored in the refrigerator compared to the other treatments. The sucrose content of the apple juice from ‘Golden Delicious’ stored unexposed to light and exposed to light was the same. Similar results were reported by Mezey and Meyezová [26], where a loss of 60.5% of the sucrose content in apple juice was observed after 140 days of cold storage. We suggest that the decrease in the sucrose content was partly attributable to the inversion of sucrose into glucose and fructose due to activity of enzyme invertase [27], as the content of glucose and fructose increased after one year of storage in both cultivars. After one year, the pasteurized apple juice from the ‘Golden Delicious’ cultivar exhibited a higher overall fructose content compared to the ‘Baya Marisa’ cultivar. The sorbitol content was the same across all the treatments and between the cultivars.

The overall citric acid content was higher in the apple juice of ‘Baya Marisa’ than in the juice of the ‘Golden Delicious’, but it did not change significantly in either cultivar after pasteurization and after one year of storage in the refrigerator, unexposed to light, and exposed to light. Similar results were obtained by Farnworth et al. [28], in which no changes in the citric acid content were measured in pasteurized and unpasteurized fruit juices and during storage.

The malic acid content was higher in the fresh pasteurized and unpasteurized apple juice of ‘Baya Marisa’ than in the same treatments of ‘Golden Delicious’; no differences were found after pasteurization. The malic acid content in the apple juices of both cultivars increased after one year of storage in the refrigerator, exposed to light, and unexposed to light. These results are in agreement with the earlier findings of Rehman et al. [29], in which the highest malic acid content was found in apple juice at the end of the 30-day storage period.

The ascorbic acid content decreased after pasteurization of the fresh apple juices of both cultivars. After one year of storage, the ascorbic acid content in the apple juices of both cultivars decreased significantly. The loss of the ascorbic acid could partly be due to the heat treatment and air in the headspace of the 100 mL glass bottles during storage [30]. On the other hand, enzymes such as ascorbic acid oxidase, peroxidase, and cytochrome oxidase are also responsible for the loss of ascorbic acid [30]. The degradation of ascorbic acid by heat treatment and storage was also reported by Tikekar et al. [31].

### 3.3. Identification of Individual Phenolic Compounds

Based on the available literature and mass spectra, a comprehensive analysis identified a total of 26 distinct phenolic compounds in the unpasteurized juice, pasteurized juice, and pasteurized juice after one year of storage. Among the phenolic compounds, 20 hydroxycinnamic acid derivatives, 1 hydroxybenzoic acid derivative, 1 dihydrochalcone, 1 flavanol, and 3 anthocyanins were identified (Table 3). Fewer individual phenolic compounds were identified compared with our previous study on fresh apples of the same cultivar (Juhart, J. et al., 2022). We hypothesize that the phenolic compounds were degraded by the thermal processing and juice pressing.

The information regarding all the identified phenolic compounds in the apple juice of ‘Baya Marisa’ and ‘Golden Delicious’ cultivars are presented in Table 2.

The phenolic compounds were identified by analyzing the mass-to-charge (*m*/*z*) ratio of specific molecular fragmentation ions. In cases where standard samples for phenolic compounds were not accessible, the initial identification relied on pseudomolecular ions, specifically [M − H]^−^ and M^+^ ions. Whenever possible, comparisons were made with authentic standards (Table 3).

### 3.4. Quantification of Individual Phenolic Compounds and Total Analyzed Phenolic Content

As shown in Table 4, the phenolic compounds were identified in all the treatments for the apple juice of both cultivars, red-fleshed ‘Baya Marisa’ and white-fleshed ‘Golden Delicious’.

Among the identified phenolic groups in the apple juice, hydroxycinnamic acids were found to be the predominant group in both the red-fleshed ‘Baya Marisa’ and white-fleshed ‘Golden Delicious’ cultivars. Chlorogenic acid was identified as the most abundant among these hydroxycinnamic acids. However, it was observed that the content of the hydroxycinnamic acids decreased following the pasteurization process in both the ‘Baya Marisa’ and ‘Golden Delicious’ apple juices. We hypothesize that the decrease in the total hydroxycinnamic acid after pasteurization was due to oxidative reactions during heating [32]. Similar results were obtained by Tian et al. [33], who found a decrease in chlorogenic acid of up to 30% after pasteurization. On the other hand, an increase in the total hydroxycinnamic acid content after pasteurization was reported, which may be due to the inactivation of enzymes from the polyphenol oxidase group [34]. However, in the samples after one year of storage in the refrigerator, at room temperature unexposed to light, and at room temperature exposed to light, the total hydroxycinnamic acid content in the pasteurized ‘Golden Delicious’ apple juice increased and was higher than that of the unpasteurized ‘Golden Delicious’ apple juice. Similar results were obtained for the apple juice of the ‘Baya Marisa’ cultivar, in which the total hydroxycinnamic acid content in the refrigerated apple juice increased and was higher than in the unpasteurized fresh apple juice. The total hydroxycinnamic acid content in the ‘Baya Marisa’ apple juice stored unexposed to light and exposed to light also increased after pasteurization, although not as much as in the ‘Golden Delicious’ juice. The cumulative content of the hydroxycinnamic acids in ‘Baya Marisa’ apple juice stored unexposed to light increased to the same level as before pasteurization, while the apple juice stored exposed to light increased to a slightly higher level than the freshly pasteurized juice. The increase in the total hydroxycinnamic acid content is mainly due to the increase in the chlorogenic acid. We suggest that our results may be due to the release of chlorogenic acid from the cell walls into the food matrix [35], since cloudy apple juice contains particles from the pulp of apple fruits (sediments), which are known to contain the highest amounts of hydroxycinnamic acids [17]. Our results are partly in agreement with those of Deng et al. [10], in which the HTST treatment did not decrease the content of the phenolic compounds in apple juice during storage. The total hydroxycinnamic acid content was higher in the samples stored in the refrigerator, which is consistent with the results of Zhao et al. [36], in which the chlorogenic acid content was lower in the samples stored at higher temperatures.

The only hydroxybenzoic acid identified was the protocatechuic acid derivative in the apple juice of red-fleshed ‘Baya Marisa’. No hydroxybenzoic acid was found in the apple juice of ‘Golden Delicious’. The total hydroxybenzoic acid content decreased after pasteurization and further decreased after one year of storage unexposed to light and exposed to light. However, the total hydroxybenzoic acid content increased significantly after one year of storage in the refrigerator. As reported by Patras et al. [24], anthocyanins can be hydrolyzed by acids during processing and storage and, moreover, can be cleaved into hydroxybenzoic acids. Hydroxybenzoic acids are known to be degradation products of anthocyanins with antimicrobial activity in processed foods [37]. We suspect that the total hydroxybenzoic acid content is partly due to the processing and degradation of the anthocyanins, as it was not detected in our previous study on fresh apples of the same cultivar [38]. The higher content of the hydroxybenzoic acid in the refrigerator-stored apple juice from ‘Baya Marisa’ suggests that the anthocyanins were cleaved into hydroxybenzoic acid. However, these results do not agree with the color measurements, as the percent red of the stored samples was highest in the refrigerator-stored samples of ‘Baya Marisa’.

Phloretin-2-*O*-xyloside was the only dihydrochalcone detected. The total dihydrochalcone content did not change significantly after pasteurization in either the ‘Baya Marisa’ and ‘Golden Delicious’ apple juice. The total dihydrochalcone content increased in all the storage methods for the pasteurized ‘Golden Delicious’ apple juice and for the ‘Baya Marisa’ apple juice stored in the refrigerator and unexposed to light, while the total dihydrochalcone content did not change in the ‘Baya Marisa’ juice exposed to light. We suspect that the higher content of phloretin-2-*O*-xyloside in the stored pasteurized apple juices of ‘Golden Delicious’ and in the refrigerator-stored apple juices of ‘Baya Marisa’ could be due to the release of this phenolic compound from small peel and pulp particles in the cloudy apple juice [6], from which phenols were released. As reported by Salazar-Orbea et al. [35], heat treatments and comminution increase the release of phenols from the peel and pulp. Our results are in partial agreement with those of Salazar-Orbea et al. [35], who reported that in some apple juices of different cultivars, the total dihydrochalcone content increased from 8% to 767% during processing and storage.

The only flavanol detected in the apple juices of ‘Baya Marisa’ and ‘Golden Delicious’ was the epicatechin derivative. The content of the epicatechin derivative decreased after pasteurization of both the apple juices of the selected cultivars. However, the content of the epicatechin derivative increased after one year of storage in the apple juices of ‘Baya Marisa’ stored in the refrigerator and unexposed to light, while the content of epicatechin derivatives in the apple juice of ‘Baya Marisa’ stored exposed to light did not change after pasteurization. Similar results were obtained for the pasteurized apple juice of ‘Golden Delicious’, in which the content of the epicatechin derivative did not change in the samples exposed to light, while it slightly increased in the samples stored in the refrigerator and unexposed to light. The low level of flavanols could be due to the destruction of the integrity of the cells during processing (pressing, mixing), which increases the exposure of flavanols to environmental factors, such as oxygen, light, enzymes, and interactions with other biomacromolecules, since this phenolic group is known to have low stability under various environmental conditions [39]. In addition, procyanidin B2 is converted by intermolecular coupling to procyanidin A2, which can be depolymerized by processing to phenolic acids. The stability of flavanols depends on the type of processing, which leads to thermal degradation, depolymerization, and polymerization [39].

As expected, anthocyanins were detected only in the red-fleshed and red-peeled apple juice of the ‘Baya Marisa’. A comparison of the anthocyanins in the fresh unpasteurized juice, fresh pasteurized juice, and pasteurized juice stored for one year showed that this group of phenolic compounds was the most unstable, as the total anthocyanin content decreased significantly after pasteurization (heat treatment) and after one year of storage in the refrigerator, unexposed to light, and exposed to light. Similar results were reported by Knebel et al. [40], in which the content of anthocyanins in depectinized apple juice of the red-fleshed cultivar ‘Maggy’ decreased significantly after processing. Our results suggest that anthocyanin degradation is associated with a higher storage temperature, as the apple juice of ‘Baya Marisa’ stored in the refrigerator had a higher anthocyanin content than the apple juice stored unexposed to light and exposed to light, indicating the higher stability of anthocyanins in a colder environment.

The anthocyanins detected in the fresh, unpasteurized, and pasteurized apple juice were cyanidin-3-arabinoside, cyanidin-3-galactoside, and peonidin-3-galactoside. Only cyanidin-3-galactoside was found in the stored apple juice from ‘Baya Marisa’ with a significantly lower content. The interaction between the fourth ring (C4) of anthocyanins and the ascorbic acid could lead to color loss in different fruit juices, since the presence of ascorbic acid in anthocyanin-containing solutions can accelerate color loss by degradation. The result of this process is the absence of the original color expression of the parental pigments [41].

**Figure 2:**
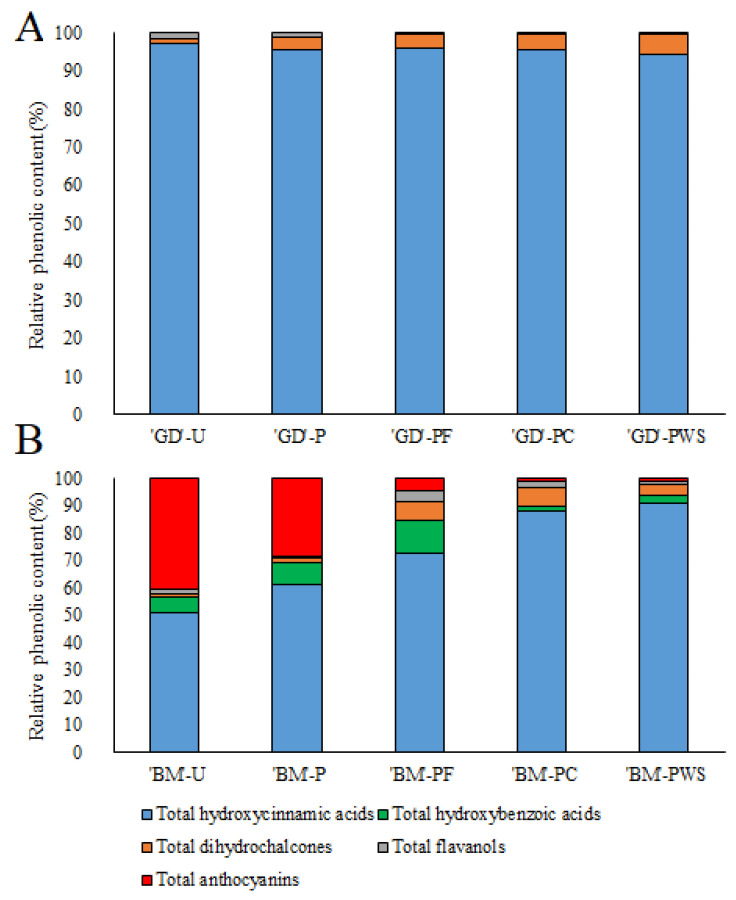
Identified phenolic compounds presented in relative content in fresh unpasteurized, pasteurized, and pasteurized and stored for one year apple (*Malus domestica* Borkh.) juice of ‘Golden Delicious’ (**A**) and ‘Baya Marisa’ (**B**). ‘BM’-U (‘Baya Marisa’ unpasteurized), ‘GD’-U (‘Golden Delicious’ unpasteurized), ‘BM’-P (‘Baya Marisa’ pasteurized), ‘GD’-P (‘Golden Delicious’ pasteurized), ‘BM’-PF (‘Baya Marisa’ pasteurized, refrigerator-stored), ‘GD’-PF (‘Golden Delicious’ pasteurized, refrigerator-stored), ‘BM’-PC (‘Baya Marisa’ pasteurized, unexposed to light), ‘GD’-PC (‘Golden Delicious’ pasteurized, unexposed to light), ‘BM’-PWS (‘Baya Marisa’ pasteurized, exposed to light), ‘GD’-PWS (‘Golden Delicious’ pasteurized, exposed to light).

The most abundant group of phenolic compounds were hydroxycinnamic acids, as shown in Figure 2.

The TAPC value was higher in the fresh, unpasteurized juice of ‘Baya Marisa’ than in ‘Golden Delicious’. The TAPC value of the apple juice for both ‘Baya Marisa’ and ‘Golden Delicious’ decreased significantly after pasteurization. However, the TAPC value of ‘Golden Delicious’ apple juice increased after one year of storage in the refrigerator, unexposed to light, and exposed to light, and was higher than that of the fresh apple juice before pasteurization. For the ‘Baya Marisa’ apple juice, the TAPC value also increased in the refrigerator and slightly increased for the apple juice unexposed to light, while the apple juice exposed to light showed the smallest decrease after one year of storage compared to the fresh juice after pasteurization. The TAPC value of the pasteurized ‘Baya Marisa’ apple juice stored in the refrigerator increased to the same TAPC value as before pasteurization. The ‘Baya Marisa’ apple juice stored in the refrigerator showed a minimal increase in the TAPC value, which was slightly higher than the fresh apple juice after pasteurization.

Overall, the TAPC value of the fresh, unpasteurized, pasteurized, and stored pasteurized apple juice of both apple cultivars was significantly lower compared to the fresh apples of these cultivars [38].

Our results show that apple juice has significantly lower levels of hydroxycinnamic acids, dihydrochalcones, flavanols, flavonols, and anthocyanins in comparison to fresh apples of the same cultivar, which were evaluated in a previously conducted study. However, hydroxybenzoic acid was detected in the apple juice, but was not found in the fresh apples of these cultivars. Higher or consistent levels of some phenolic compounds in the apple juices after one year of storage could be due to the pectin present in cloudy apple juice, as it has a protective effect by forming colloidal suspensions that limit the degradation of phenolic compounds [35].

## 4. Conclusions

The TAPC and sugar and organic acid content were found to be lower overall in the apple juices of both cultivars than in fresh apples of the same cultivars. For consumers, this means that they ingest fewer phenolic compounds overall with juice pressed from the fresh apple.

The total hydroxycinnamic acid content decreased after pasteurization of the juices of both cultivars and increased after one year of storage in all the treatments, except in the RME samples of cultivar ‘Baya Marisa’, compared to freshly pasteurized apple juice.

Hydroxybenzoic acids were detected only in the cultivar ‘Baya Marisa’; however, the total hydroxybenzoic acid content decreased after pasteurization, then decreased even further after one year of storage, except for the refrigerator-stored samples, in which the total hydroxybenzoic acid content increased compared to the freshly pasteurized apple juice.

The total dihydrochalcone content remained stable after pasteurization in the apple juices of both cultivars and increased after a year of storage compared to the freshly pasteurized apple juice of both cultivars, except in the RME samples of the cultivar ‘Baya Marisa’.

The total flavanol content decreased after pasteurization in both apple juices and remained the same after a year of storage, except in the refrigerated samples and the RMU samples of the ‘Baya Marisa’ apple juice, in which the total flavanol content increased compared to the freshly pasteurized samples.

The total anthocyanin content decreased after pasteurization, then decreased even further after one year of storage, which indicates the high instability of this group of phenolic compounds.

Different storage methods had different effects on the color parameters, suggesting the influence of the storage technique on the preservation of the original color, which is one of the most important factors in the food industry.

The results of this study show that the best storage location for apple juice is the refrigerator, as TAPC content is highest after one year of storage. The lowest levels were measured in samples of both apple juices exposed to light at room temperature. Our results also suggest that phenolic compound preservation is cultivar-dependent. In addition, studies on enzyme activity and air exposure should be carried out, as they affect the degradation, polymerization, and depolymerization of secondary metabolites and, thus, the interactions of primary and secondary metabolites.

## Figures and Tables

**Table 1:** The color parameters (represented as mean ± standard error) of apple juice derived from the red-fleshed ‘Baya Marisa’ and white-fleshed ‘Golden Delicious’ apple cultivars were assessed both at the time of harvest and following one year of storage.

Cultivar	Color Intensity	% Yellow	% Red	% Blue	Tonality
‘Baya Marisa’ non-pasteurized	5.54 ± 0.01 a	43.17 ± 0.02 i	41.27 ± 0.04 b	15.57 ± 0.00 g	1.05 ± 0.00 h
‘Baya Marisa’ pasteurized	5.43 ± 0.01 b	43.12 ± 0.01 i	41.52 ± 0.01 a	15.63 ± 0.00 f	1.04 ± 0.00 h
‘Baya Marisa’ refrigerated	2.82 ± 0.00 g	44.37 ± 0.03 h	40.12 ± 0.03 c	15.52 ± 0.00 g	1.11 ± 0.00 g
‘Baya Marisa’ unexposed to light	3.88 ± 0.02 d	51.49 ± 0.09 e	33.87 ± 0.04 e	14.64 ± 0.12 h	1.52 ± 0.00 e
‘Baya Marisa’ exposed to light	5.33 ± 0.01 c	47.48 ± 0.01 g	34.67 ± 0.01 d	17.85 ± 0.00 b	1.37 ± 0.00 f
‘Golden Delicious’ non pasteurized	3.14 ± 0.00 e	55.72 ± 0.02 a	28.18 ± 0.01 j	16.10 ± 0.01 e	1.98 ± 0.00 a
‘Golden Delicious’ pasteurized	3.11 ± 0.01 e	55.61 ± 0.01 b	28.26 ± 0.01 i	16.13 ± 0.01 e	1.97 ± 0.00 a
‘Golden Delicious’ refrigerated	3.06 ± 0.00 f	51.22 ± 0.01 f	29.74 ± 0.00 f	19.04 ± 0.01 a	1.72 ± 0.00 d
‘Golden Delicious’ unexposed to light	2.22 ± 0.01 i	55.44 ± 0.02 c	28.41 ± 0.01 h	16.15 ± 0.01 d	1.95 ± 0.00 b
‘Golden Delicious’ exposed to light	2.44 ± 0.00 h	53.36 ± 0.01 d	29.02 ± 0.00 g	17.62 ± 0.01 c	1.84 ± 0.00 c

Statistically significant differences are presented with different letters after the mean values within the same columns. Same letters after the mean values in the same columns indicates no statistically significant differences.

**Table 2:** Individual sugars and organic acids (mean ± SE, in g/L) in unpasteurized, freshly pasteurized, and pasteurized apple juice stored for one year in a refrigerator, unexposed to light, and exposed to light, of cultivars ‘Baya Marisa’ and ‘Golden Delicious’.

	‘Baya Marisa’					‘Golden Delicious’				
Compounds	Fresh	Pasteurization	Refrigerator	Unexposed to Light	Exposed to Light	Fresh	Pasteurization	Refrigerator	Unexposed to Light	Exposed to Light
Sugars										
Sucrose	43.11 ± 5.94 a	42.81 ± 4.02 a	5.19 ± 0.30 d	4.38 ± 0.30 e	4.08 ± 0.60 e	41.19 ± 3.84 a	37.71 ± 5.22 a	21.27 ± 0.60 b	8.67 ± 0.06 c	8.58 ± 0.15 c
Glucose	20.97 ± 1.77 b	21.57 ± 1.59 b	25.17 ± 0.66 a	25.53 ± 0.87 a	25.95 ± 0.57 a	23.94 ± 2.82 b	23.58 ± 1.17 b	26.4 ± 0.18 a	28.83 ± 0.11 a	29.79 ± 0.15 a
Fructose	56.31 ± 0.33 c	58.51 ± 0.93 c	67.26 ± 0.33 b	66.93 ± 0.96 b	65.07 ± 0.22 b	68.04 ± 0.36 b	65.85 ± 0.33 b	73.5 ± 0.39 a	71.16 ± 0.60 a	69.54 ± 0.69 a
Sorbitol	6.93 ± 1.11 a	7.08 ± 0.66 a	15.76 ± 0.57 a	5.67 ± 0.54 a	5.76 ± 0.51 a	6.36 ± 2.31 a	6.27 ± 0.57 a	5.70 ± 0.57 a	5.70 ± 0.60 a	5.82 ± 0.57 a
Organic acids										
Citric acid	6.03 ± 0.87 a	5.73 ± 0.36 a	8.16 ± 1.77 a	7.05 ± 0.48 a	6.93 ± 0.36 a	4.74 ± 0.81 b	4.35 ± 0.57 b	5.37 ± 0.36 b	4.77 ± 0.72 b	5.01 ± 1.71 b
Malic acid	37.5 ± 4.98 b	32.94 ± 5.43 b	62.85 ± 3.24 a	58.80 ± 3.36 a	54.96 ± 3.48 a	26.67 ± 3.06 c	24.96 ± 3.27 c	57.87 ± 3.57 a	56.97 ± 3.21 a	56.64 ± 3.18 a
Ascorbic acid	0.018 ± 0.003 a	0.012 ± 0.003 b	0.003 ± 0.000 c	0.003 ± 0.000 c	0.003 ± 0.000 c	0.015 ± 0.003 a	0.009 ± 0.003 b	0.003 ± 0.000 c	0.003 ± 0.000 c	0.003 ± 0.000 c

Different letters between treatments (within the same rows) following mean values are significantly different (*p* < 0.05).

**Table 3:** Tentative identification of the 26 individual phenolic compounds of apple juices of *Malus domestica* Borkh. cultivars ‘Baya Marisa’ and ‘Golden Delicious’ and used standards.

Phenolic Compounds	Rt (min)	[M − H]^−^ (m/z)	M^+^ (*m/z*)	MS^2^ (*m/z*)	MS^3^ (*m/z*)	Expressed as
**Hydroxycinnamic acids**						
*p*-coumaric acid hexoside derivative 1	10.2	371		325,163		*p*-coumaric acid
*p*-coumaric acid hexoside derivative 2	12.3	371		325,163		*p*-coumaric acid
*p*-coumaric acid hexoside 1	14.2	325		265,235,163		*p*-coumaric acid
Caffeic acid derivative	16.9	295		251,189,179	179,135	caffeic acid
Caffeic acid derivative 2	12.9	311		179		caffeic acid
Caffeic acid hexoside 1	10.6	341		179,135		caffeic acid
Dicaffeic acid derivative 1	10.8	457		277,185,179	179,135	caffeic acid
Dicaffeic acid derivative 2	19.3	403		233,179	179,135	caffeic acid
Dicaffeic acid derivative 3	22.6	429		249,205,179		caffeic acid
Dicaffeic acid derivative 4	24.3	473		429,249,179	249,205,179	caffeic acid
Dihydrodicaffeic acid derivative	20.5	405		225,181		caffeic acid
4-*O*-*p*-Coumaroylquinic acid	16.2	337		173,163		chlorogenic acid
5-*O*-*p*-Coumaroylquinic acid	17.1	337		191,163,119		chlorogenic acid
Chlorogenic acid (5-caffeoylquinic acid)	13.4	353		191,179		chlorogenic acid
Caffeoylferuoylquinic acid	13.9	563		517,385,321,179	205,191,103	chlorogenic acid
Ferouoylquinnic acid gallate	10.9	658		466,385,273	193,191	chlorogenic acid
Feruloylquinic acid derivative 1	12.7	431		385,331	193,191	chlorogenic acid
Feruoylquinnic acid hexoside	20.9	547		385,235	193,191	chlorogenic acid
Ferulic acid hexoside derivative	11.6	401		355	193	ferulic acid
Cryptochlorogenic acid (4-caffeoylquinic acid)	15.3	353		191,179		cryptochlorogenic acid
**Hydroxybenzoic acids**						
Protocatechuic acid derivative	14.2	481		327,153	153,109	protocatechuic acid
**Dihydrochalcones**						
Phloretin-2-*O*-xyloside	22.2	567		273,167		phloridzin
**Flavanols**						
(epi)catechin derivative	22.1	579		289,271,245,205		(-) epicatechin
**Anthocyanins**						
Cyanidin-3-*O*-galactoside	8.9		449	287		cyanidin-3-*O*-galactoside
Cyanidin-3-*O*-arabinoside	11.9		419	287		cyanidin-3-*O*-arabinoside
Peonidin-3-*O*-galactoside	11.0		463	301		peonidin-3-*O*-galactoside

Rt, retention time; [M − H]^−^ pseudomolecular ion identified in a negative ion mode; M^+^ , pseudomolecular ion identified in a positive ion mode.

**Table 4:** Phenolic compounds in unpasteurized and pasteurized apple juice of both apple (*Malus domestica* Borkh.) cultivars ‘Baya Marisa’ and ‘Golden Delicious’ (mean ± SE, in mg/L juice) immediately after pressing and after one year of storage in a refrigerator, at room temperature unexposed to light (RMU), and at room temperature exposed to light (RME).

	‘Baya Marisa’					‘Golden Delicious’				
Compounds	Fresh	Pasteurization	Refrigerator	RMU	RME	Fresh	Pasteurization	Refrigerator	RMU	RME
Hydroxycinnamic acids										
*p*-coumaric acid hexoside derivative 1	1.82 ± 0.01 a	0.85 ± 0.02 b	1.04 ± 0.34 b	0.25 ± 0.34 c	0.39 ± 0.15 c	nd	nd	nd	nd	nd
*p*-coumaric acid hexoside derivative 2	1.27 ± 0.03 a	1.00 ± 0.03 b	0.73 ± 0.09	0.24 ± 0.08	0.76 ± 0.14	nd	nd	nd	nd	nd
*p*-coumaric acid hexoside 1	0.25 ± 0.01 c	0.17 ± 0.04 c	1.02 ± 0.12 a	0.46 ± 0.19 b	0.43 ± 0.18 b	0.48 ± 0.07 b	0.36 ± 0.08 b	0.71 ± 0.14 a	0.74 ± 0.10 a	0.92 ± 0.25 a
Caffeic acid derivative	2.07 ± 0.11 a	1.23 ± 0.21 b	0.91 ± 0.16 c	0.56 ± 0.14 d	0.49 ± 0.09 d	0.99 ± 0.11 c	0.25 ± 0.01 e	1.47 ± 0.20 b	1.40 ± 0.17 b	0.71 ± 0.13 c
Caffeic acid derivative 2	12.1 ± 0.03 a	9.44 ± 0.02 b	nd	nd	nd	0.7 ± 0.03 c	0.7 ± 0.01 c	nd	nd	nd
Dicaffeic acid derivative 1	0.39 ± 0.02 a	0.27 ± 0.03 b	nd	nd	nd	0.22 ± 0.01 a	0.19 ± 0.01 a	nd	nd	nd
Dicaffeic acid derivative 2	0.96 ± 0.07 c	0.85 ± 0.09 c	1.94 ± 0.24 a	1.03 ± 0.11 c	0.35 ± 0.09 d	0.83 ± 0.07 c	0.68 ± 0.09 b	1.02 ± 0.04 b	0.86 ± 0.11 c	0.54 ± 0.09 d
Dicaffeic acid derivative 3	0.48 ± 0.02 b	0.39 ± 0.01 b	1.78 ± 0.25 a	0.89 ± 0.02 b	0.14 ± 0.01 c	0.47 ± 0.09 b	0.31 ± 0.00 b	0.57 ± 0.04 b	1.53 ± 0.07 a	0.29 ± 0.02 c
Dicaffeic acid derivative 4	0.23 ± 0.05 a	0.16 ± 0.03 b	nd	nd	nd	0.37 ± 0.09 a	0.19 ± 0.02 b	0.21 ± 0.02 b	0.15 ± 0.02 c	0.14 ± 0.01 c
Caffeic acid hexoside 1	1.66 ± 0.07 a	0.88 ± 0.03 b	nd	nd	nd	nd	nd	nd	nd	nd
Dihydrodicaffeic acid derivative	0.30 ± 0.02 c	0.28 ± 0.04 c	1.57 ± 0.07 a	0.56 ± 0.05 b	0.34 ± 0.02 c	0.36 ± 0.01 c	0.37 ± 0.00 c	0.47 ± 0.06 c	0.41 ± 0.03 c	0.63 ± 0.03 b
4-*O*-*p*-Coumaroylquinic acid	1.00 ± 0.03 c	0.55 ± 0.03 d	3.39 ± 0.20 a	1.80 ± 0.30 b	1.77 ± 0.27 b	0.41 ± 0.05 d	0.35 ± 0.03 d	2.23 ± 0.25 ab	1.90 ± 0.32 b	1.17 ± 0.20 b
5-*O*-*p*-Coumaroylquinic acid	0.08 ± 0.01 d	0.09 ± 0.01 d	2.47 ± 0.10 c	2.37 ± 0.73 c	1.03 ± 0.08 c	6.29 ± 0.03 b	1.75 ± 0.05 c	14.62 ± 0.65 a	12.51 ± 0.49 a	6.52 ± 0.45 b
Chlorogenic acid (5-caffeoylquinic acid)	37.08 ± 0.18 e	16.00 ± 0.11 f	67.56 ± 2.68 d	54.70 ± 1.77 e	36.00 ±1.97 e	55.47 ± 0.04 d	13.99 ± 0.08 f	124.49 ± 6.58 a	109.59 ± 4.87 b	94.29 ± 4.19 c
Caffeoylferuoylquinic acid	1.40 ± 0.11 b	0.76 ± 0.12 c	2.26 ± 0.35 a	1.83 ± 0.26 b	0.74 ± 0.17 c	1.67 ± 0.01 b	0.56 ± 0.05 c	1.37 ± 0.18 bc	1.66 ± 0.25 b	1.71 ± 0.33 b
Ferouoylquinnic acid gallate	4.84 ± 0.06 b	3.40 ± 0.08 c	2.96 ± 0.49 c	1.26 ± 0.11 d	1.5 ± 0.48 d	8.07 ± 0.02 a	6.46 ± 0.13 b	7.81 ± 0.19 b	5.98 ± 1.01 b	3.80 ± 0.71 c
Feruloylquinnic acid derivative 1	3.06 ± 0.08 a	2.91 ± 0.12 a	3.75 ± 0.79 a	4.15 ± 0.91 a	3.64 ± 0.89 a	0.94 ± 0.01 b	0.85 ± 0.04 b	1.45 ± 0.15 a	1.67 ± 0.24 a	1.73 ± 0.10 a
Ferulic acid hexoside derivative	0.35 ± 0.02 c	0.18 ± 0.03 d	1.57± 0.19 a	0.69 ± 0.18 b	0.27 ± 0.09 c	0.33 ± 0.01 c	nd	nd	nd	nd
Feruoylquinic acid hexoside	1.68 ± 0.02 a	1.29 ± 0.02 b	0.78 ± 0.09 c	0.41 ± 0.10 cd	0.13 ± 0.01 d	0.83 ± 0.07 c	0.52 ± 0.05 c	0.63 ± 0.09 c	0.64 ± 0.07 c	0.70 ± 0.09 c
Cryptochlorogenic acid (4-caffeoylquinic acid)	3.34 ± 0.05 a	2.51 ± 0.04 b	2.49 ± 0.07 b	0.68 ± 0.15 d	0.82 ± 0.04 d	1.47 ± 0.07 c	1.00 ± 0.04 c	1.17 ± 0.14 c	1.01 ± 0.04 c	0.94 ± 0.03 c
Hydroxybenzoic acids										
Protocatechic acid derivative	8.53 ± 0.09 b	5.66 ± 0.08 c	17.01 ± 0.93 a	1.54 ± 0.24 d	1.53 ± 0.24 d	nd	nd	nd	nd	nd
Dihydrochalcones										
Phloretin-2-*O*-xyloside	1.52 ± 0.25 d	1.02 ± 0.09 d	10.70 ± 0.13 a	5.74 ± 0.28 b	2.47 ± 0.37 c	1.01 ± 0.02 d	0.99 ± 0.04 d	5.96 ± 0.68 b	6.50 ± 0.48 b	6.69 ± 0.75 b
Flavanols										
(epi)catechin derivative	2.54 ± 0.09 b	0.52 ± 0.03 d	5.43 ± 0.23 a	2.11 ± 0.24 b	0.55 ± 0.12 d	1.26 ± 0.01 c	0.35 ± 0.03 d	0.78 ± 0.03 dc	0.66 ± 0.18 dc	0.55 ± 0.06 d
Anthocyanins										
Cyanidin-3-arabinoside	0.81 ± 0.13 a	0.58 ± 0.09 a	nd	nd	nd	nd	nd	nd	nd	nd
Cyanidin-3-galactoside	58.17 ± 0.19 a	19.07 ± 0.08 b	4.80 ± 0.25 c	0.50 ± 0.02 d	0.36 ± 0.04 d	nd	nd	nd	nd	nd
Peonidin-3-galactoside	0.90 ± 0.16 a	0.41 ± 0.01 b	nd	nd	nd	nd	nd	nd	nd	nd
Total hydroxycinnamic acids	74.39 ± 0.59 c	43.18 ± 0.58 de	106.90 ± 7.23 b	74.21 ± 1.89 c	51.01 ± 0.67 d	79.89 ± 0.10 c	28.49 ± 0.45 e	162.35 ± 6.75 a	148.26 ± 1.56 a	122.40 ± 2.00 b
Total hydroxybenzoic acids	8.53 ± 0.09 a	5.66 ± 0.08 b	17.01 ± 0.93 a	1.54 ± 0.24 d	1.53 ± 0.24 d	nd	nd	nd	nd	nd
Total dihydrochalcones	1.52 ± 0.25 c	1.02 ± 0.09 c	10.70 ± 0.13 a	5.74 ± 0.28 b	2.47 ± 0.37 c	1.01 ± 0.02 c	0.99 ± 0.04 c	5.96 ± 0.68 b	6.50 ± 0.48 b	6.69 ± 0.75 b
Total flavanols	2.54 ± 0.09 b	0.52 ± 0.03 d	5.43 ± 0.23 a	2.11 ± 0.24 b	0.55 ± 0.12 d	1.26 ± 0.01 c	0.35 ± 0.00 d	0.78 ± 0.03 dc	0.66 ± 0.18 dc	0.55 ± 0.06 d
Total anthocyanins	59.88 ± 0.38 a	20.07 ± 0.13 b	4.80 ± 0.10 c	0.50 ± 0.04 d	0.35 ± 0.01 d	nd	nd	nd	nd	nd
TAPC	146.86 ± 0.93 bc	70.45 ± 0.43 de	146.65 ± 8.20 bc	84.46 ± 1.78 d	56.18 ± 1.04 e	82.16 ± 0.08 d	29.83 ± 0.42 f	169.08 ± 6.08 a	155.42 ± 1.45 ab	129.64 ± 2.62 c

The data presented in the table are expressed as means ± standard error. Different letters are used to indicate significant differences (*p* < 0.05) among the means within rows for unpasteurized, pasteurized, and stored pasteurized apple juice kept in the refrigerator, at room temperature unexposed to light, and at room temperature exposed to light. The abbreviation “nd” indicates that the component was not detected.

## Data Availability

The data presented in this study are available on request from the corresponding author.
